# Length-change patterns of the medial collateral ligament and posterior oblique ligament in relation to their function and surgery

**DOI:** 10.1007/s00167-020-06050-0

**Published:** 2020-06-01

**Authors:** Lukas Willinger, Shun Shinohara, Kiron K. Athwal, Simon Ball, Andy Williams, Andrew A. Amis

**Affiliations:** 1 The Biomechanics Group, Department of Mechanical Engineering, Imperial College London, London, SW7 2AZ UK; 2 Department of Orthopaedic Sports Medicine, Technical University of Munich, Hospital Rechts Der Isar, Munich, Germany; 3 Faculty of Engineering, Hokkaido University, Sapporo, 060-8628 Japan; 4 Fortius Clinic, 17 Fitzhardinge St, London, W1H 6EQ UK; 5 Musculoskeletal Surgery Group, Imperial College London School of Medicine, London, W6 8RF UK

**Keywords:** Medial collateral ligament, Posterior oblique ligament, Length change, Reconstruction, Isometry

## Abstract

**Purpose:**

To define the length-change patterns of the superficial medial collateral ligament (sMCL), deep MCL (dMCL), and posterior oblique ligament (POL) across knee flexion and with applied anterior and rotational loads, and to relate these findings to their functions in knee stability and to surgical repair or reconstruction.

**Methods:**

Ten cadaveric knees were mounted in a kinematics rig with loaded quadriceps, ITB, and hamstrings. Length changes of the anterior and posterior fibres of the sMCL, dMCL, and POL were recorded from 0° to 100° flexion by use of a linear displacement transducer and normalised to lengths at 0° flexion. Measurements were repeated with no external load, 90 N anterior draw force, and 5 Nm internal and 5 Nm external rotation torque applied.

**Results:**

The anterior sMCL lengthened with flexion (*p* < 0.01) and further lengthened by external rotation (*p* < 0.001). The posterior sMCL slackened with flexion (*p* < 0.001), but was lengthened by internal rotation (*p* < 0.05). External rotation lengthened the anterior dMCL fibres by 10% throughout flexion (*p* < 0.001). sMCL release allowed the dMCL to become taut with valgus rotation (*p* < 0.001). The anterior and posterior POL fibres slackened with flexion (*p* < 0.001), but were elongated by internal rotation (*p* < 0.001).

**Conclusion:**

The structures of the medial ligament complex react differently to knee flexion and applied loads. Structures attaching posterior to the medial epicondyle are taut in extension, whereas the anterior sMCL, attaching anterior to the epicondyle, is tensioned during flexion. The anterior dMCL is elongated by external rotation. These data offer the basis for MCL repair and reconstruction techniques regarding graft positioning and tensioning.

## Introduction

The medial ligament structures of the knee, including the superficial medial collateral ligament (sMCL), the deep medial collateral ligament (dMCL), and the posterior oblique ligament (POL) within the posteromedial capsule (PMC), work together to restrain valgus and internal/external rotatory loads [27, 28, 33]. The MCL is the most commonly injured ligament of the knee and ruptures may occur either in isolation or in combination with damage to other ligamentous or meniscal structures [1]. Non-surgical treatment, principally bracing, rest, and rehabilitation, is the primary treatment for grade I- and II-MCL strains. However, a proportion of grade III injuries, implying a rupture of the three MCL structures [24], knees with persistent valgus instability despite non-surgical treatment [25, 35], and some dMCL lesions with chronic pain [21] require surgery.

Many MCL reconstruction techniques have been proposed [6], which implies that none is ideal. However, due to inconsistent anatomical descriptions, reconstruction techniques differ in the number and the site of attachment points, the number of graft bundles, and graft tensioning protocol [3, 15, 19, 20, 23]. These differences reflect a lack of knowledge of the attachment sites and the resulting length-change patterns of the natural and reconstructed soft tissues. Perhaps, because of this, the present authors find the MCL the most difficult of the knee ligaments to reconstruct reliably. Registry data [31] have found that when an MCL injury in association with ACL rupture is treated conservatively, the likelihood of ACL graft failure is increased. These observations suggest the importance of improving the treatment of medial soft-tissue injuries.

Although injuries of the dMCL contribute significantly to anteromedial rotatory instability (AMRI) [14, 29], established MCL reconstruction techniques do not restore the function of the dMCL. Furthermore, some previous biomechanical tests of MCL reconstruction have been performed, while the dMCL was intact, so they could not have reproduced the grade III injury pattern that is usually required to indicate surgery [5]. A correct understanding of the behaviour of the medial knee structures would profoundly help surgeons to improve surgical techniques for treating medial instability.

The purpose of this study was to define the length-change patterns of the medial knee structures (sMCL, dMCL, POL) at their anterior and posterior borders, across the knee flexion range and with applied anterior and rotational loads.

It was hypothesised that:

1. Structures attaching anteriorly and inferiorly to the medial epicondyle (anterior sMCL, anterior dMCL) [28] would lengthen with flexion, and structures inserting posteriorly to the medial epicondyle (posterior sMCL, posterior dMCL, and POL) would be tight in extension and slacken with flexion.
2. The sMCL and the dMCL would be lengthened by external rotation, and the POL by internal rotation. Anterior drawer force was not expected to cause significant length changes in the MCL complex with the anterior cruciate ligament (ACL) intact.

## Materials and methods

Following (Imperial College Healthcare Tissue Bank) ethics approval, ten unpaired fresh-frozen human cadaveric knees (8 male and 2 female) with an average age of 61 (range 51–69) years were used. All specimens were stored at − 20 °C and were thawed 24 h at room temperature before preparation. They were confirmed to have no evidence of previous surgery, nor abnormal laxity, nor misalignment by visual and manual examination by an orthopaedic surgeon (LW). Knees were kept moist with occasional water spray during the entire test.

### Specimen preparation

The femur and tibia were cut 20 cm and 15 cm from the joint line, respectively. The fibula was cut and secured to the tibia in its anatomical position by a transcortical bone screw. Skin and subcutaneous fat were removed leaving the muscles, cruciate and collateral ligaments, and the capsule intact. The thigh muscles were divided into their anatomical parts and cloth strips were sutured to the proximal muscle ends to allow tension to be applied via free-hanging weights connected to the muscles by a string and pulley system. The load distribution was based on the muscle physiological cross-sectional areas [7] as in the previous reports [17, 30]; the total muscle tension of 60 N ensured that a tibiofemoral joint contact force of approximately 30 N was imposed over the entire flexion/extension arc, eliminating separation between the articular surfaces.

**Fig. 1:**
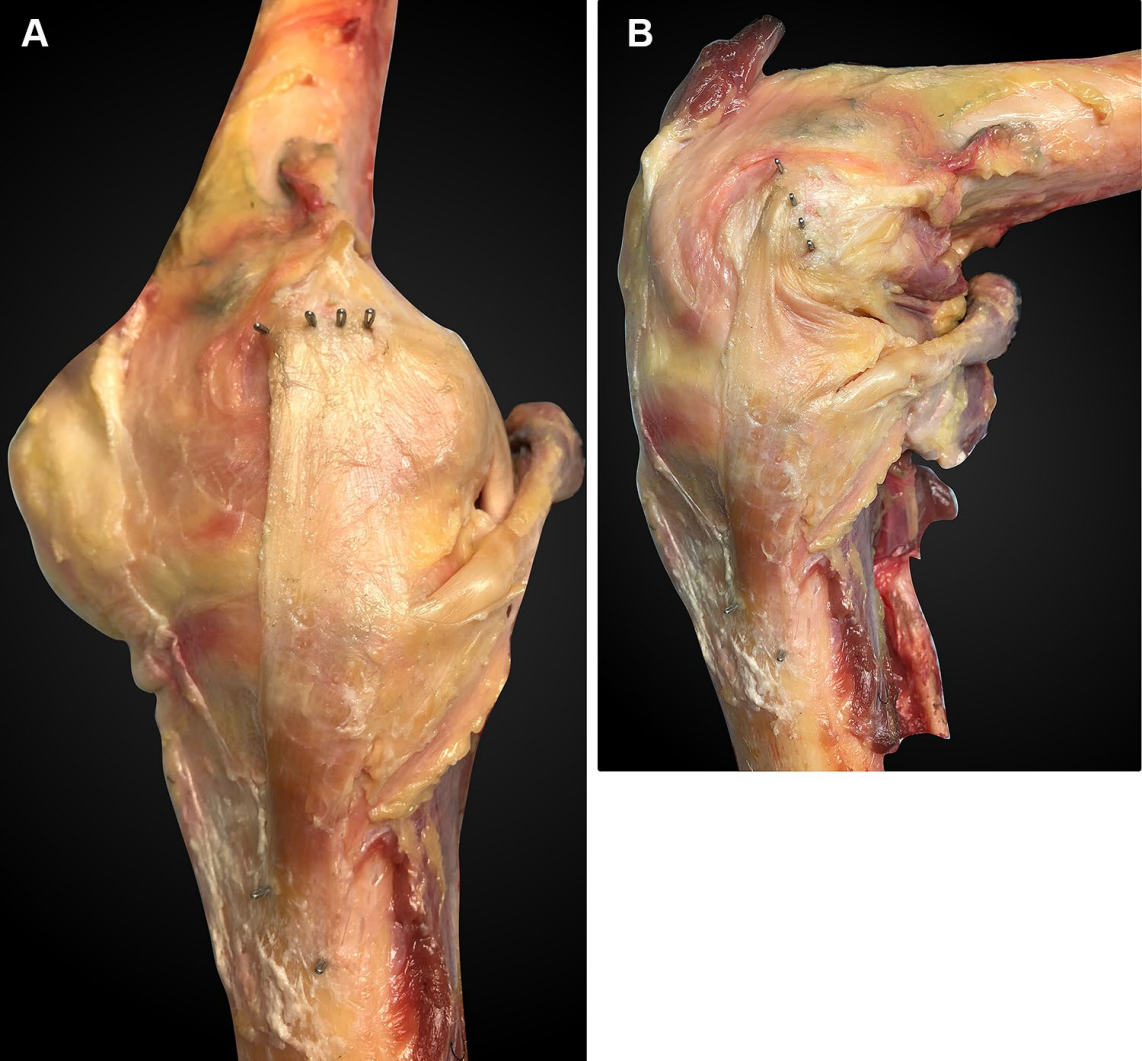
Medial aspect of a right knee: **a** near extension and **b** at 90° flexion. The four staples in the femur are, from anterior to posterior: the anterior edge of the sMCL, the posterior edge of the sMCL, the anterior edge of the POL, and the posterior edge of the POL. They were hammered completely into the bone after attaching a suture to each of them. The staple loops which guided the anterior and posterior sMCL sutures are visible distally, at the sMCL tibial attachment. The anterior margin of the sMCL wraps around the femoral medial epicondyle with knee flexion. The femoral medial epicondyle is located midway between the anterior and posterior fibre attachments of the sMCL. Note that the distal staples of the POL, in the posterior rim of the tibial plateau, are obscured by the semimembranosus tendon

The knee was flexed and extended to observe the tightening-slackening behaviour and thereby to identify the anterior and posterior borders of the sMCL (Fig. 1). The border between the sMCL and the PMC was identified by a difference in fibre orientations: the sMCL being essentially proximal to distal, whilst the PMC fibres were oblique, coursing in a distal–posterior direction from femur to tibia. The POL fibres were located subjectively within the width of the PMC. The PMC fibres attaching to the semimembranosus tendon were removed to reveal the course of the POL to its bony attachment at the posteromedial rim of the proximal tibia. The connection between the sMCL and the PMC was kept intact to avoid alterations of the kinematic behaviour.

It was difficult to observe the dMCL without disturbing the fibres of the overlying sMCL. Therefore, to gain access to the dMCL without damaging the sMCL, a tibial bone block (of average dimensions 45 mm proximal–distal length × 28 mm anterior–posterior width × 14 mm medial–lateral depth) with the distal sMCL attached was elevated by saw-cut osteotomy. Two holes were pre-drilled through the bone block and tibia prior to the osteotomy to ensure subsequent correct repositioning using two bicortical bone screws, and 1 mm-thick spacers were inserted to account for the thickness of the saw cuts. The sMCL with the tibial bone block was dissected sharply from the underlying tissue and reflected from distal to proximal; this included disruption of the flimsy proximal tibial attachment deep to the sMCL [18]. To check whether the osteotomy and reflection of the tibial sMCL attachment had an effect on sMCL length-change measurements, a preliminary test was undertaken on five specimens. The lengths of the anterior and posterior fibres of the sMCL were measured across the arc of knee flexion both before and after the bone block was created and the sMCL reflected, and the repeated measurements compared.

**Fig. 2:**
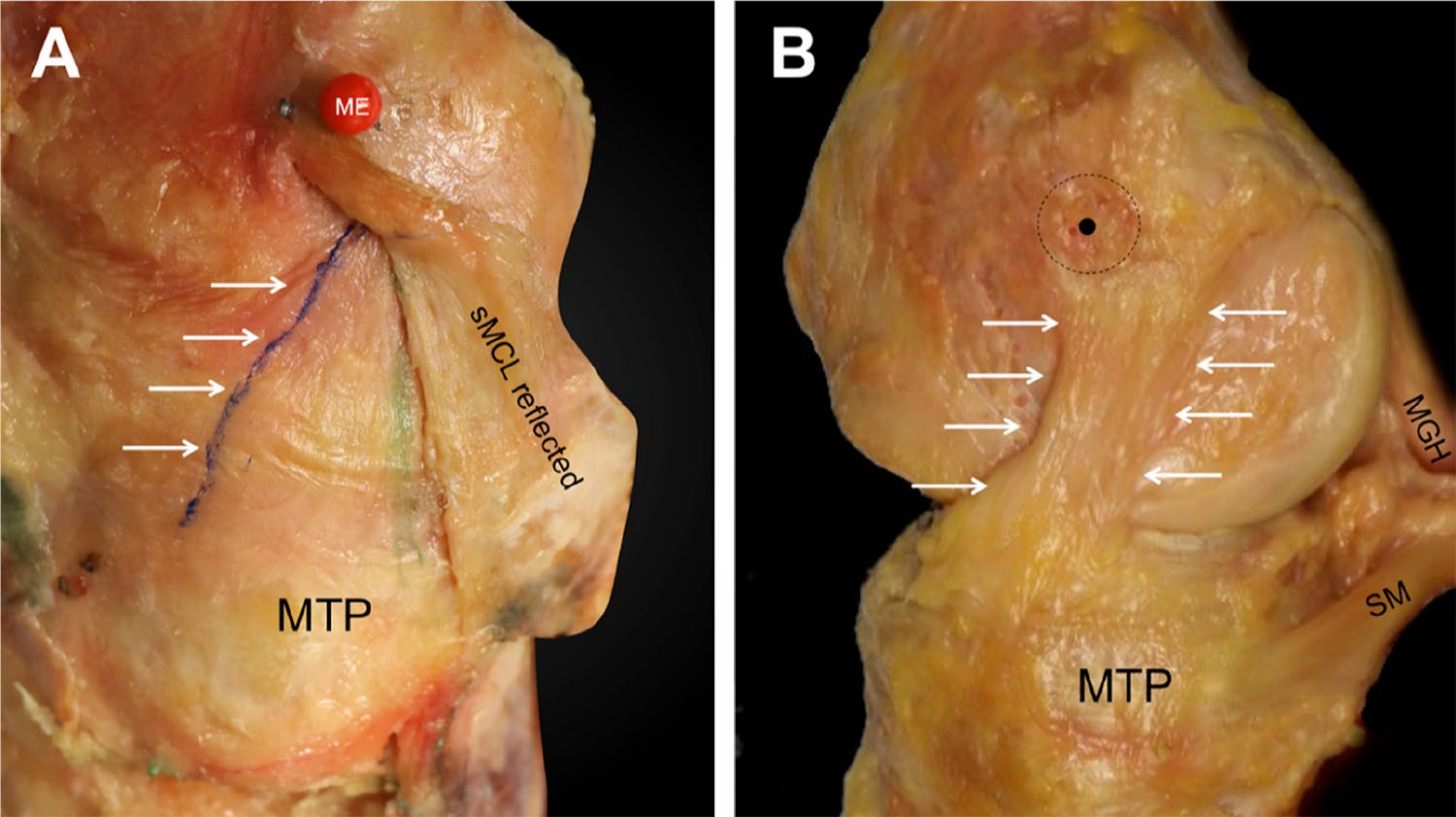
**a** The dMCL of an extended right knee, anterior to the left of the picture and the long axis of the tibia vertically downwards. The red pin head is at the most prominent point of the femoral medial epicondyle (ME): the sMCL attaches anterior and posterior to it. The sMCL femoral attachment is intact, showing the anterior fibres passing anterior to the epicondyle. Distally, the sMCL has been reflected posteriorly (but not released completely) as far as the green line where the sMCL and dMCL blend together to form the PMC, revealing the dMCL, with its most-posterior fibres marked by the green line. The femoral attachment of the dMCL is distal and posterior to the epicondyle, so it is obscured by the sMCL. The most-anterior fibres of the dMCL—the blue line highlighted by the arrows—are oriented anterior/distal from the femur to the tibia. The wrinkle/buckling across the width of the dMCL indicates that it is slack when the sMCL is intact. MTP: medial tibial plateau. **b** The sMCL has been removed: the most prominent point of the medial epicondyle (black dot) is at the centre of the sMCL attachment (black dashed circle). The oblique antero-distal orientation of the dMCL in neutral tibial rotation is evident, with the anterior and posterior borders indicated by the white arrows. *SM* direct head of semimembranosus muscle, *MGH* medial head of gastrocnemius muscle, *MTP* medial tibia plateau

The dMCL was exposed as far posterior as the merging of layers 2 and 3 of the medial soft tissues with the PMC as previously described [28, 34] (Fig. 2). The femoral and tibial attachments of the dMCL were identified and their fibre behaviour observed during the flexion/extension arc. The femoral and tibial attachment points of the sMCL, dMCL, and POL were marked with small metal staples (6 mm long × 1 mm wide) inserted into the femur and tibia at the respective anterior and posterior borders of each structure.

**Fig. 3:**
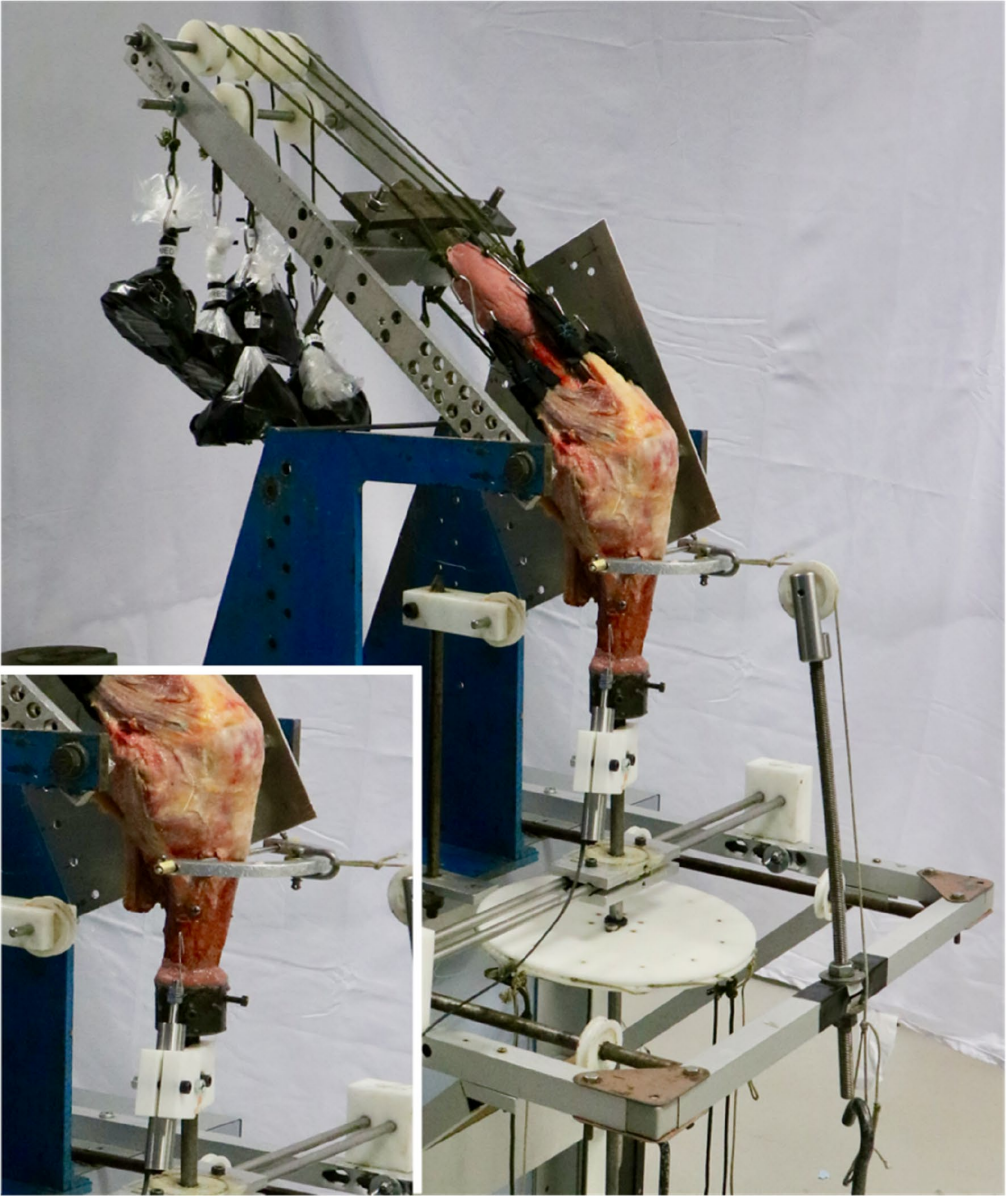
The knee was secured in a 6-DOF kinematics rig which allowed testing across knee flexion between 0° and 100° and applying anterior draw and rotational loads. Muscle loads were applied by hanging weights via a cord and pulley system. A linear variable displacement transducer was mounted alongside the tibial rod with a customised 3D-printed fixture (see inset)

Poly-methyl methacrylate bone cement was used to fix an intramedullary rod into the femur and to embed the distal tibia into a pot with a 50 cm axial extension rod. The femoral intramedullary rod was fixed to a 6 degree-of-freedom (DOF) kinematic rig with the femur in its anatomical 6° valgus offset, so that the tibia was vertical. The epicondylar axis of the femur was aligned parallel to the floor and corresponded to the rotation axis of the rig. The rig allowed passive motion of the femur from 0° to 100° flexion, while the tibia hung vertically and uninhibited (Fig. 3).

### Measurements

A linear variable displacement transducer (LVDT) (30 mm stroke LVDT, Solartron Metrology, UK; linearity < 0.3%) was mounted alongside the tibial rod with a 3D-printed fixture. A monofilament stainless steel suture (# 5–0; diameter 0.12 mm) was attached to each of the femoral staples, routed along the edge of the ligament and through the respective tibial staple and collinearly connected to the LVDT. The monofilament suture slid freely upon the tissue without being stretched and thereby enabling length-change measurements between the femoral and tibial staples across the investigated range of motion (ROM). The suture was tensed by the weight of the sliding core of the LVDT (0.6 N). The length changes were recorded for every 10° of knee flexion. Measurements were repeated three times and the average was taken for analysis. In this study, for brevity and consistency, we use ‘lengthening’ to mean that the distance between the femoral and tibial attachments of a ligament fibre has increased, and vice versa for ‘shortening’. That is not the same as elastic stretching or tensioning of the ligament fibre itself—it is possible to have a ‘lengthening’ measured between the attachments, while the fibre remains slack, for example. This study is concerned with the length-change patterns between the attachments, not the ligament tensions.

To apply external loads, a 5.5 mm Steinmann pin was drilled from medial to lateral through the proximal tibia and a semicircular metal hoop was mounted on it. The hoop was used to apply 90 N anterior draw force by a string, pulley, and hanging weights without constraining internal–external rotation. Additionally, a 250 mm polyethylene disc was fixed to the tibial extending rod to allow the application of 5 Nm internal and external rotational torques using a string and pulley system (Fig. 3).

The datum length of each soft-tissue structure was measured with a flexible ruler to an accuracy of ± 0.5 mm at 0° knee flexion. The length-change data across knee flexion and with applied loads were then normalised to the fibre length at 0° flexion:

$$ {\text{Strain}} = \left( {{\text{change in fibre length}}/{\text{fibre length at }}0^\circ } \right) \times {1}00\% . $$

To measure the medial joint line opening (i.e., a valgus laxity measure) between an intact sMCL and a deficient sMCL, two pins were placed—one fixed into the most distal part of the medial femoral condyle and another to the most proximal part of the medial tibia, with their heads protruding immediately anterior to the fibres of the sMCL and dMCL. Changes in the length between them reflected opening/closing of the medial joint. The specimens were rigidly mounted horizontally with the knee in full extension and the medial aspect uppermost. The length between the femoral and tibial pins was measured using a Vernier caliper (to ± 0.1 mm) with the tibial and femoral condyles in contact. The sMCL was then released by elevating the tibial bone block. The tibia was then freed from its mounting and allowed to drop into valgus under its own weight without further external applied loads, and this increased the length between the femoral and tibial pins, which was remeasured. This process was performed with the valgus opening movement of the tibia constrained in internal–external rotation (that is: an isolated valgus motion), then repeated with the tibia allowed to externally rotate under the influence of its weight, while it dropped into valgus. These measurements were repeated at 0° and 30° knee flexion.

### Statistical analysis

Data were analysed using SPSS statistics software version 24.0 (IBM, New York, USA). Normal distribution was confirmed by the Shapiro–Wilk test. For each structure tested (anterior and posterior fibres of the sMCL, dMCL, and POL, respectively), two-way repeated-measures analyses of variance (ANOVA) were performed to compare the normalised length changes (dependent variable) to the external load applied (unloaded, anterior translation force, internal rotation torque, and external rotation torque) across different flexion angles. A one-way repeated-measures ANOVA was performed for each structure to compare length change across flexion angles with the initial measure at 0° knee flexion. When differences were found, paired t tests with Bonferroni correction were performed. Statistical significance was set at *p* < 0.05.

## Results

The preliminary test of five specimens confirmed that the osteotomy of the tibial sMCL attachment had no significant effect on sMCL length-change measurements. It revealed a maximum length-change deviation of 0.4% for the anterior sMCL at 100° and 0.6% for the posterior sMCL at 80° of knee flexion (n.s.) compared to the knees prior to elevating the bone block.

The native length of each structure at 0° knee flexion was: 111 ± 6 mm for the anterior sMCL, 117 ± 6 mm for the posterior sMCL, 40 ± 4 mm for the anterior and posterior dMCL, 46 ± 4 mm for the anterior POL, and 48 ± 6 mm for the posterior POL.

### Anterior superficial MCL

**Fig. 4:**
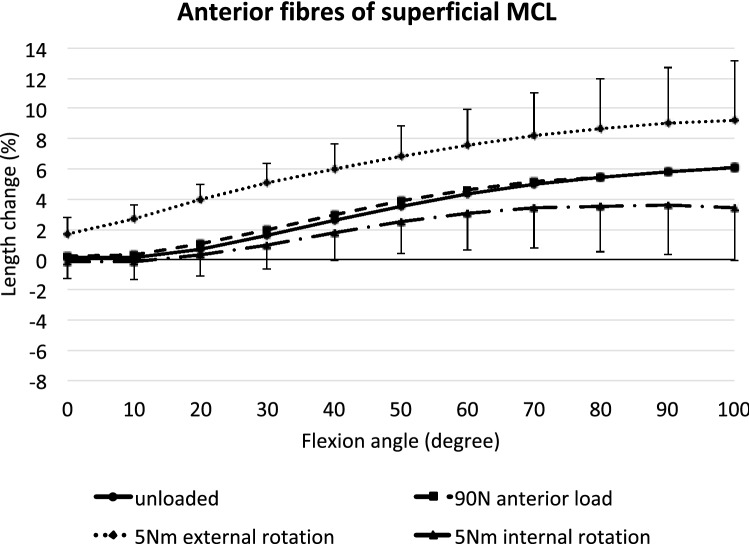
Length changes of the anterior fibres of the superficial MCL across knee flexion, with the tibia unloaded and with anterior translation force, internal rotation torque, and external rotation torque applied to the tibia. Shown as mean values with ± SD; *n* = 10. Significant length changes are described in the text

Without external load, the anterior fibres of the sMCL were increasingly lengthened with increasing flexion angle from 30° to 100° (*p* < 0.01) (Fig. 4). In addition, external rotation significantly lengthened these fibres compared to the unloaded condition (*p* < 0.05 at full extension and *p* < 0.001 from 10 to 100° flexion). An anterior translation force had no significant effect. Tibial internal rotation led to a significant shortening of the anterior fibres of the sMCL between 80° and 100° knee flexion (*p* < 0.05).

### Posterior superficial MCL

**Fig. 5:**
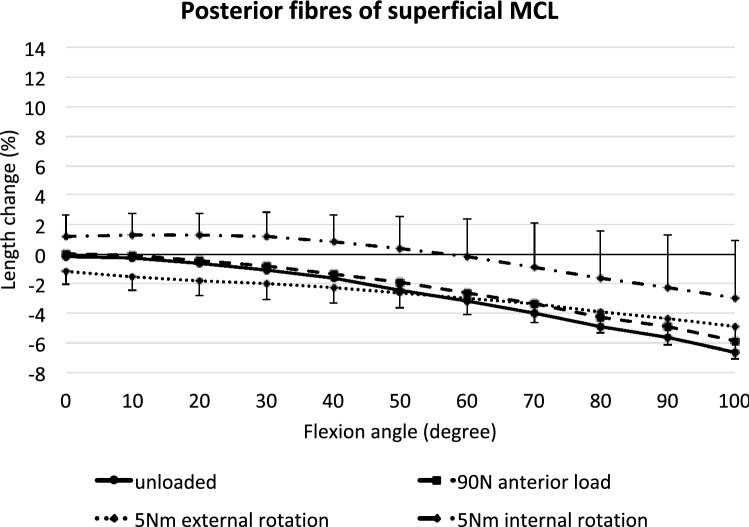
Length changes of the posterior fibres of the superficial MCL across knee flexion, with the tibia unloaded and with anterior translation force, internal rotation torque, and external rotation torque applied to the tibia. Shown as mean values with ± SD; *n* = 10. Significant length changes are described in the text

In contrast to the anterior fibres, the posterior fibres of the sMCL continuously shortened with knee flexion (*p* < 0.05 from 30 to 50°, *p* < 0.001 from 60 to 100°) (Fig. 5). An anterior tibial translation force did not affect the length-change patterns of the posterior sMCL compared to the unloaded condition (n.s.). External rotation shortened the fibres significantly close to full extension (*p* < 0.05 at 0° and 10°), while internal rotation lengthened these fibres (*p* < 0.05 between 10° and 100°).

### Anterior deep MCL

**Fig. 6:**
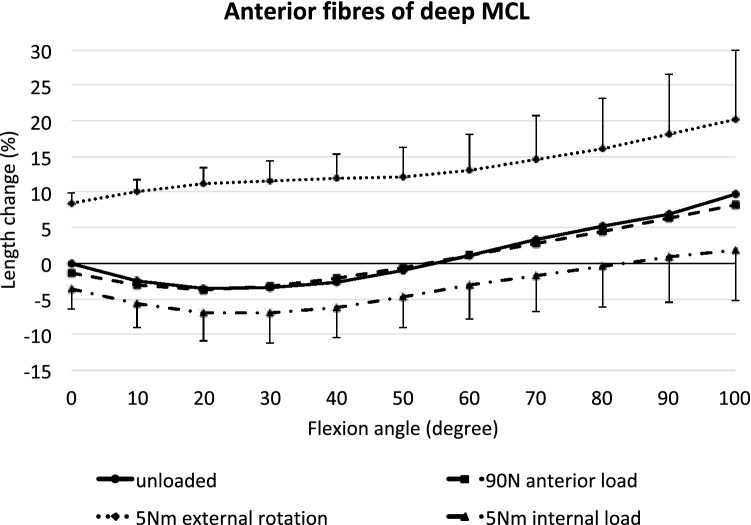
Length changes of the anterior fibres of the deep MCL across knee flexion, with the tibia unloaded and with anterior translation force, internal rotation torque, and external rotation torque applied to the tibia. Shown as means with ± SD; *n* = 10. Significant length changes are described in the text

The anterior fibres of the dMCL shortened significantly from 0° to 30° knee flexion (*p* < 0.05) and lengthened from 60° to deeper flexion, but without reaching statistical significance (Fig. 6). An anterior translation force only significantly slackened the fibres at full extension (*p* < 0.05), but even this was a very small length change (< 0.5 mm). Tibial external rotation led to a large lengthening of 9–20% compared to the unloaded state across the observed range of movement (*p* < 0.001). Internal rotation shortened the anterior dMCL significantly at 0° and 10° as well as between 50° and 100° (*p* < 0.05).

### Posterior deep MCL

**Fig. 7:**
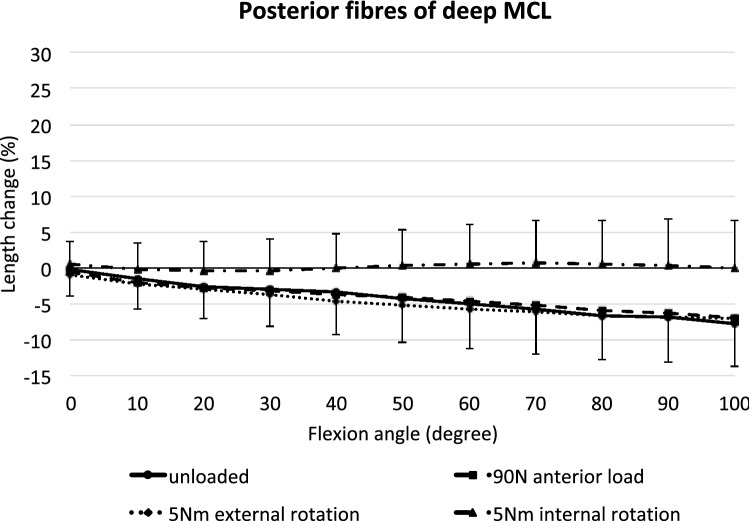
Length changes of the posterior fibres of the deep MCL across knee flexion, with the tibia unloaded and with anterior translation force, internal rotation torque, and external rotation torque applied to the tibia. Shown as mean values with ± SD; *n* = 10. Significant length changes are described in the text

The posterior fibres of the dMCL shortened increasingly from 0° to 100° flexion (*p* < 0.05; Fig. 7). Tibial internal rotation significantly reduced this phenomenon between 50° and 100° of flexion (*p* < 0.05), while anterior translation force and external rotation torque did not significantly affect the length of the posterior dMCL compared to the unloaded knee.

During the preliminary study of the effect of creating the distal sMCL bone block, it was observed that when the knee was in the flexion range 0°–30° flexion, the whole of the sMCL was taut, but that when the tibial attachment of the sMCL was elevated with the bone block and its proximal weak tibial attachment was released, the dMCL was revealed and was clearly slack, along with the joint capsule (Fig. 2a). That created a valgus laxity, which, with valgus stress, was abolished, thereby causing the dMCL to become taut.

After releasing the sMCL, the distance between the femoral and tibial marker pins (which were at the anterior fibres of the dMCL in the extended knee) increased by 2.6 ± 1.9 mm under valgus load of 0.5 Nm with constrained internal–external rotation, and 3.2 ± 2.1 mm with unrestricted external rotation at 0° knee flexion (both, *p* < 0.01). At 30° knee flexion, the distance increased by 3.4 ± 0.9 mm under valgus load with constrained internal–external rotation, and by 5.0 ± 1.8 mm with unrestricted external rotation (both, *p* < 0.001). The slack in the dMCL, whilst the sMCL is intact, has never previously been reported.

### Anterior POL

**Fig. 8:**
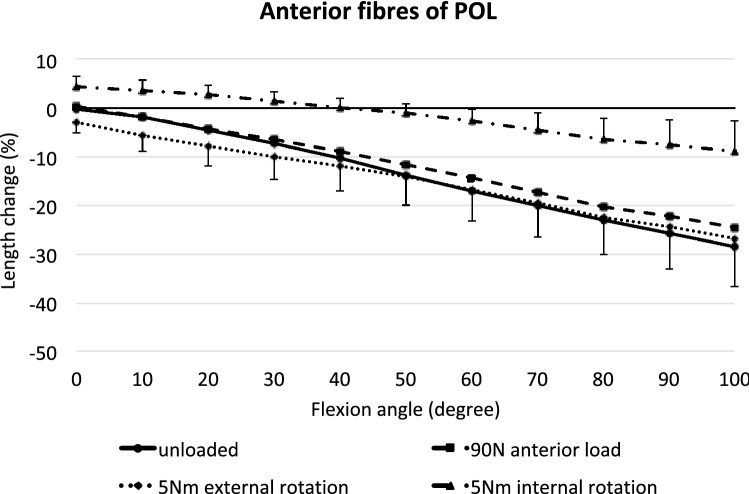
Length changes of the anterior fibres of the POL across knee flexion, with the tibia unloaded and with anterior translation force, internal rotation torque, and external rotation torque applied to the tibia. Shown as means with ± SD; *n* = 10. Significant length changes are described in the text

The anterior fibres of the POL shortened with knee flexion (*p* < 0.01 at 10° and *p* < 0.001 from 20° to 100°), reaching 28% shortening at 100° flexion (Fig. 8). Anterior translation force, or external rotation torque, had very small and variable effects on anterior POL length (*p* < 0.05 for anterior translation only at 60° flexion, and *p* < 0.05 for external rotation at full extension). Tibial internal rotation, however, resulted in a significant lengthening at all flexion angles compared to the unloaded condition (*p* < 0.001). This effect increased with knee flexion, reaching 18% lengthening caused by internal rotation at 100° flexion. However, due to the shortening with knee flexion, internal rotation did not return the fibres to their datum length beyond 40° knee flexion.

### Posterior POL

**Fig. 9:**
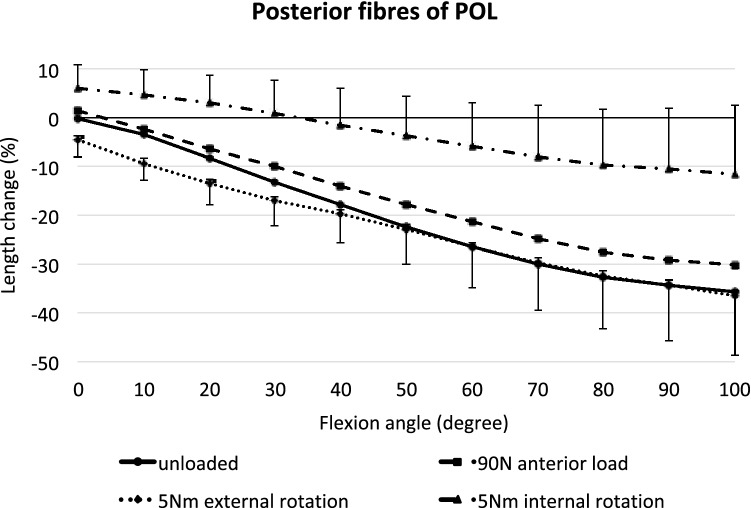
Length changes of the posterior fibres of the POL across knee flexion, with the tibia unloaded and with anterior translation force, internal rotation torque, and external rotation torque applied to the tibia. Shown as means with ± SD; *n* = 10. Significant length changes are described in the text

The posterior fibres of the POL became progressively shorter with knee flexion, and then were visibly very slack (up to 35% shortening) (*p* < 0.01 at 10° and *p* < 0.001 from 20° to 100°; Fig. 9). An anterior translation force tended to lengthen the fibres in the flexed knee (*p* < 0.05 at 50°, 60°, and 80–100°), while a tibial external rotation tended to shorten the posterior POL near knee extension (*p* < 0.05 at 0°–10°), but these were small effects, typically a mean change of 5% fibre length. An applied internal rotation torque lengthened the posterior POL fibres (*p* < 0.05 from 0° to 20° and *p* < 0.001 in deeper flexion). As with the anterior POL fibres, this effect was much greater than the length changes induced by the other loads, reaching a mean lengthening of 24% at 100° flexion, and did not return the posterior POL fibres to their datum length above 32° knee flexion.

## Discussion

The most important finding of this study was that the ligaments at the medial aspect of the knee differ in their length-change responses to knee flexion, and to external applied loads. The MCL and POL are not isometric—the situation is complex. In particular, structures attaching posterior to the medial epicondyle—the posterior fibres of the sMCL, the posterior fibres of the dMCL, and both the anterior and posterior fibres of the POL—shorten/slacken with increasing knee flexion. Conversely, the anterior fibres of the sMCL lengthen during knee flexion, since they are attached anterior to the axis of knee flexion, which is at the medial epicondyle, as had been hypothesised. The anterior part of the dMCL is slack in early flexion, since its femoral attachment is distal and posterior to the medial epicondyle and lengthens in deeper flexion once its femoral attachment moves relative to the flexion axis. Application of an external rotation torque to the tibia significantly lengthens the anterior fibres of the dMCL throughout the flexion arc. The anterior and posterior fibres of the POL slacken rapidly with knee flexion, while tibial internal rotation lengthens them, but the rotation effect does not cancel the slackening that occurs with knee flexion except near to knee extension. These length changes with tibial external and internal rotation supported the initial hypotheses of the present study.

A novel finding is that when the sMCL is intact and the knee is near extension, the dMCL and adjacent joint capsule are slack in neutral tibial rotation; releasing the sMCL leads to approximately 3 mm joint line opening at 0° and 5 mm 30° knee flexion with a valgus load applied. In addition, whilst most descriptions of dMCL anatomy suggest a vertical alignment in the sagittal plane, the present study describes, for the first time, that the dMCL passes distally and anteriorly to create obliquity in its alignment which is ideal for resisting tibial external rotation.

These descriptive findings can be applied to the clinical focus on how best to restore normal stability and function to the injured knee, in particular with AMRI, which may also be associated with ACL injury.

As the knee flexes, the femoral attachment of the sMCL rotates around the medial epicondyle, resulting in a lengthening of the anterior fibres and a shortening of the posterior fibres. The strain patterns of the sMCL fibres were, therefore, primarily dependent on the femoral attachment site with regard to the centre of rotation at the medial epicondyle. An anterior translation (draw) force did not affect the sMCL length-change patterns, while the ACL, the primary restraint, was intact. However, the anterior sMCL fibres were lengthened by tibial external rotation, while the posterior fibres were lengthened by internal rotation. These findings agree with the previous studies that have similarly described different length-change patterns depending on the femoral sMCL attachment sites [2, 9, 13, 22, 32, 33]. Arms et al. [2], using a strain transducer, found the highest strain at the proximal anterior sMCL fibres as the knee flexed and a decrease of strain at the posterior fibres at the same time. A similar strain pattern was reported by Gardiner et al. [10] who used optical strain measurement. Furthermore, fluoroscopic studies in vivo agreed with these findings and described an elongation of the anterior part of the sMCL [13, 22]. In contrast with earlier works and the present study, Feeley et al. [8] reported a slackening with flexion for all MCL graft attachment points studied. They also reported the sMCL attachment to be posterior to the medial epicondyle, as did Laprade et al. [18], which differs from the present observations (Figs. 1 and 2). Victor et al. [32] found that an MCL fibre attached proximal and posterior to the medial epicondyle was near-isometric, whereas the reciprocal length-change patterns of the anterior and posterior fibres of the sMCL in this present study indicate that the epicondyle will be isometric.

The results of the present study are very relevant to clinical practice. Regarding sMCL reconstruction techniques, the present data suggest that a femoral attachment at the centre of the anatomical attachment, enveloping the medial epicondyle, would lead to the most isometric behaviour (Fig. 1). Graft positioning posterior to the medial epicondyle, as recommended by other authors and commonly practiced [10, 19], would lead to a slackening of the graft with knee flexion, and thereby be likely to compromise clinical results.

The femoral attachment of the dMCL is just distal and slightly posterior to the sMCL attachment and its fibres fan out to its wider tibial attachment. This present work emphasizes that the anterior fibres of the dMCL run anterodistally from the femur to the anteromedial tibia (Fig. 2). This oblique orientation means that the dMCL is well oriented to withstand tibial external rotation, and the fibres can be clearly identified under tension, whilst tibial external rotation is applied. The shape of the dMCL and the obliquity of the anterior portion have not been described previously. Earlier anatomical studies, which have usually shown the dMCL as having a purely proximal–distal orientation in the sagittal plane may have described the posterior portion—the oblique anterior fibres become clear when the tibia is in external rotation. The present results showed a large lengthening strain of the anterior dMCL when the tibia was externally rotated. It was also observed that in neutral tibial rotation, the dMCL was loose, while the sMCL was intact, with an average of 5.0 mm slack at 30° knee flexion. We hypothesise that this dMCL laxity might reduce the risk of dMCL rupture, given that its short fibres can withstand less elongation to failure than those of the longer sMCL [26], and accounts for the dMCL’s main function of resisting external rotation [4, 11, 27] and not valgus stress, against which the sMCL provides the most important restraint [12]. It also explains the minimal contribution of the dMCL to restraint of valgus load, while the sMCL and PMC are intact [27]. These findings suggest that dMCL injuries may occur through a mechanism involving tibial external rotation [14], which also corresponds clinically to dMCL injury [21]. Presumably, since the dMCL and sMCL are commonly injured together a combination of external rotation plus valgus can be the injurious mechanism as well as major pure valgus. In the sporting scenario, the latter mechanism is common in rugby and American football but the former in football (soccer) [21] and alpine skiing.

To date, acute dMCL injuries are solely treated non-surgically and no reconstruction technique has been described. However, isolated injuries of the dMCL lead to a significant increase of tibial external rotation near knee extension, and could, therefore, possibly result in AMRI of the knee [4, 14, 29], particularly in footballers who impose tibial external rotation loads with certain kicking techniques and sudden change of direction. In footballers, dMCL injuries can lead to failure of healing and disabling pain, which does require surgery at times [21]. Typically, in this scenario, the original injury is relatively minor allowing rapid return to activity such as running, but perhaps at the cost of inhibiting healing, and development of an inflamed region of dMCL—usually close to the femoral attachment. The footballers concerned only have pain when the dMCL is stressed. Hence usually they ‘can do everything except- “bend” a ball’. To do that, the player’s foot addresses the ball in a way that jerks the knee into external rotation. It is a manoeuvre that a player uses so frequently that the pain associated with it is disabling. If the dMCL injury is noted on MRI, it is, therefore, good to advise the player and medical team to rehabilitate more slowly avoiding external rotational load (for example, walking for 4 weeks with the foot straight ahead rather than the natural 10 degrees or so of external rotation during stance phase of gait), and most do heal. However, persistence of symptoms at 12 weeks may be a reason to offer surgery, which involves exposure of the dMCL lesion (including splitting what appears to be a normal sMCL), its suture repair and a layer-by-layer closure of the sMCL (layer 2 [34], and deep fascia (layer 1 [34]).

The posteromedial capsule (PMC) includes fibres that are often described as the posterior oblique ligament (POL). The anterior POL fibres slacken immediately with knee flexion, and the posterior fibres slacken even more, demonstrating that the more-posterior the femoral attachments, the greater the slackening with knee flexion. This accords with the previous findings that the main contribution of the POL is to resist valgus loading at and near full knee extension [27]. The present work has found that the POL becomes taut when tibial internal rotation is applied at flexion angles up to 40°, beyond which the slackening with knee flexion causes it to be ineffective. Hence, the POL inhibits tibial internal rotation of the extended knee [27]. Although a knee trauma sustained close to extension including an internal rotation torque could injure the PMC/POL, it is a rare scenario where the POL is injured in isolation rather than with the PCL, for example.

The results of this study suggest that a POL reconstruction should be tensioned and fixed in full knee extension and neutral rotation to reproduce its anatomical length, and prevent over-tightening. Previous reconstruction techniques [15, 20] tensioned the POL graft at 30° and 60° knee flexion, respectively, but that would not recreate POL anatomical function. It would risk inability to fully extend the knee, and a fixed flexion deformity is a cause of great morbidity.

This study has limitations. The effect of applying a valgus moment to the knee was not studied despite resistance to valgus angulation being the primary restraining action of the MCL, leading to direct MCL elongation [12]. The applied muscle loads were only a small portion of physiological loads; they were used to maintain joint contact for the length-change measurements, and not to simulate a specific load-bearing activity. Although the sMCL only showed a flimsy attachment to the proximal tibia, disrupting it to access the dMCL could have led to small alterations in biomechanical behaviour, but it had no effect on the length patterns of the fibres of the sMCL. Similarly, the osteotomy of the distal tibial attachment of the sMCL might have altered its position when replaced for subsequent testing. This study was the first that investigated in vitro length-change patterns of the dMCL, while the sMCL and the PMC were intact and, hence, simulated an intact uninjured condition. The methods for length-change measurements and loading were similar to well-established test protocols [16, 30]. Length changes depend critically on the bone attachment locations, so a study of inter-and intra-observer errors of locating the femoral medial epicondyle was conducted by three of the authors across five knees: the mean inter-observer error was 1.2 ± 0.8 mm in anterior–posterior and 1.9 ± 1.3 mm proximal–distal with an overall ICC 0.851 (95% CI 0.644–0.956).

## Conclusion

The anterior and posterior fibres of the sMCL act reciprocally either side of the femoral medial epicondyle: as the knee flexes, the anterior sMCL lengthens/tightens, whilst the posterior fibres shorten/slacken. The anterior fibres of the dMCL are oriented in an oblique antero-distal direction, so they are predominantly lengthened/tensioned by tibial external rotation. The dMCL is significantly loose in neutral rotation when the sMCL is intact. The POL is tight in knee extension, and further lengthened/tightened by tibial internal rotation, but it immediately shortens/slackens with knee flexion. This knowledge is critical to optimize reconstructive surgery and to understand the aetiology of injury patterns to the medial ligamentous structures.
